# Impact of Ribosome Activity on SARS-CoV-2 LNP – Based mRNA Vaccines

**DOI:** 10.3389/fmolb.2021.654866

**Published:** 2021-04-20

**Authors:** Evangelos Tsiambas, Aristeidis Chrysovergis, Vasileios Papanikolaou, Nicholas Mastronikolis, Vasileios Ragos, Anna Batistatou, Dimitrios Peschos, Nikolaos Kavantzas, Andreas C. Lazaris, Efthimios Kyrodimos

**Affiliations:** ^1^Department of Cytology, Molecular Unit, 417 Veterans Army Hospital (NIMTS), Athens, Greece; ^2^Department of Pathology, Medical School, University of Ioannina, Ioannina, Greece; ^3^Department of Maxillofacial, Medical School, University of Ioannina, Ioannina, Greece; ^4^1st ENT Department, Hippocration Hospital, National and Kapodistrian University, Athens, Greece; ^5^ENT Department, Medical School, University of Patras, Patras, Greece; ^6^Department of Physiology, Medical School, University of Ioannina, Ioannina, Greece; ^7^Department of Pathology, Medical School, National and Kapodistrian University, Athens, Greece

**Keywords:** ribosome, mRNA, SARS-CoV-2, COVID-19, vaccine, LNP

## Abstract

Coronavirus-related Severe Acute Respiratory Syndrome-2 (SARS-CoV-2) initially was detected in Wuhan, Hubei, China. Since early 2021, World Health Organization (WHO) has declared Coronavirus Disease 2019 (COVID-19) a pandemic due to rapidly transformed to a globally massive catastrophic viral infection. In order to confront this emergency situation, many pharmaceutical companies focused on the design and development of efficient vaccines that are considered necessary for providing a level of normalization in totally affected human social-economical activity worldwide. A variety of vaccine types are under development, validation or even some of them have already completed these stages, initially approved as conditional marketing authorisation by Food and Drug Administration (FDA), European Medicines Agency (EMA), and other national health authorities for commercial purposes (*in vivo* use in general population), accelerating their production and distribution process. Innovative nucleoside-modified viral messenger RNA (v-mRNA)—based vaccines encapsulated within nanoparticles—specifically lipid ones (LNPs)—are now well recognized. Although this is a promising genetic engineering topic in the field of nanopharmacogenomics or targeted nucleic vaccines, there are limited but continuously enriched *in vivo* data in depth of time regarding their safety, efficacy, and immune response. In the current paper we expand the limited published data in the field of ribosome machinery and SARS-CoV-2 mRNA fragment vaccines interaction by describing their functional specialization and modifications. Additionally, alterations in post-transcriptional/translational molecules and mechanisms that could potentially affect the interaction between target cells and vaccines are also presented. Understanding these mechanisms is a crucial step for the next generation v-mRNA vaccines development.

## Introduction

**FIGURE 1:**
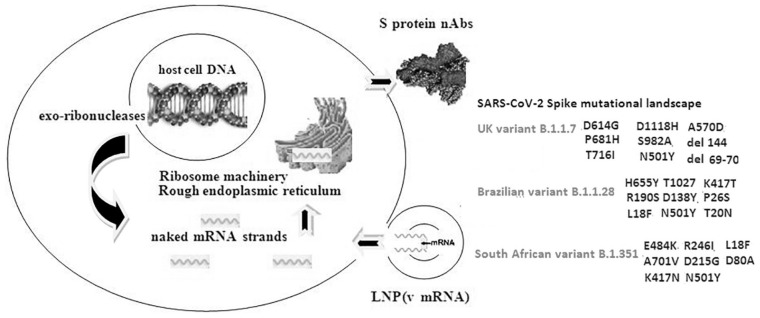
Schematic presentation of a target cell—mRNA LNP based vaccine interaction and the SARS-CoV-2 Spike glycoprotein major mutational landscape. Nucleoside-modified viral messenger RNA (v mRNA) genomic sequence is encapsulated within nanoparticles—specifically lipid ones (LNPs). Insertion of the LNP-m RNA complex is followed by translation of the corresponding transcript in the ribosome machinery and final synthesis of the S protein S protein neutralizing antibodies (nAbs) in rough endoplasmic reticulum which are exported from the cell membrane in order to sensitize T cells and motivate a specific anti SARS-CoV-2 immune response. Mutations (specific RNA substitutions and deletions) affecting the spike glycoprotein create the corresponding virus RNA variants and are responsible for different rates of anti-SARS-CoV-2 resistance reducing immune response in the first generation v-mRNA vaccines.

Coronavirus-related Severe Acute Respiratory Syndrome (SARS-CoV) in 2002/2003, Middle-East Respiratory Syndrome (MERS-CoV) in 2012/2013, and especially the current 2019/2020 Severe Acute Respiratory Syndrome-2 (SARS-CoV-2) led to an unpredictable stress regarding the national health systems’ endurance worldwide (Song et al., 2019). Rapidly global spread of Coronavirus Disease 2019 pandemic (COVID-19)—characterized by elevated infectivity and mortality—increased the need and pressure for specific anti-SARS-CoV-2 targeted therapeutic strategies *via* monoclonal antibodies (mAbs). Furthermore, massive production of safe and effective vaccines initially validated by multi-omics—based integrated analyses is another critical aspect (Barh et al., 2020). Efficient vaccines are essential for succeeding a level of normalization in totally affected human social-economical activity worldwide. A variety of vaccine types are under development in different phases, under validation or even some of them have already completed these stages and initially approved as conditional marketing authorisation by Food and Drug Administration (FDA), European Medicines Agency (EMA) and other national health authorities. Concerning their commercial *in vivo* use in general population, production, and distribution process has been already accelerated (Dai and Gao, 2020). Nucleoside-modified viral messenger RNA (v-mRNA)—based vaccines encapsulated within nanoparticles—specifically lipid ones (LNPs)—are now well recognized. Safety, efficacy, and immune response are main parameters for evaluating their quality. In fact, obvious, apparent and suspected adverse reactions (side effects) are under investigation (Flanagan et al., 2020). In the current paper we focused deeply on specific intracellular interactions with potential impact on mRNA vaccines functionality (ribosome functional specialization, modifications, alterations in post-transcriptional/translational mechanisms and other critical molecules) (Figure 1).

## Ribosome Biogenesis, Structure and Function

Structural biology evolution has revealed human ribosome (HR) biogenesis as a major and most significant event for the cellular micro-environmental homeostasis. Mature HRs are macromolecular complexes that act as intracellular network factories (translational apparatus) providing a continuous protein synthesis (Nissen et al., 2000). They are recognized as isolated domains or attached to the endoplasmic reticulum in the cytoplasm. Concerning their mature phenotype, they consist of two subunits—the large (60S) subunit and the small (40S) subunit- composing an active for translational procedure 80S HR (Pelletier and Sonenberg, 2019). A totally complete HR structure includes a ribonucleoprotein complex containing seventy-nine ribosomal proteins and four ribosomal RNAs. Analyzing each subunit, we recognize the presence of 18S rRNA and 33 proteins in the 40S, whereas 60S demonstrates 28S, 5S, and 5.8S rRNAs and also 47 proteins. RNA polymerase (RNA pol) I and III are the transcribers of these rRNAs in a sequential and multi-step procedure that drives the pre-60S and pre-40s ribosomal domains to their structurally and functionally mature formations (Kressler et al., 2010). HR biogenesis arises from specific intranuclear regions called nucleoli that surround the nucleolar organizer regions in the acrocentric chromosomes. They are characterized by a high protein density level explaining their clear visibility under the bright-field conventional microscopes. Their functional overactivation leads to rRNAs synthesis, their assembly with the corresponding proteins as a result of an energy-intensive process (Henras et al., 2015). These pre-mature domains are exported from nuclear micro-environment to the cytoplasm in order to create a massive macromolecular machinery mechanism for translating mRNAs into proteins. HRs binding to mRNAs is followed by mRNA codons transformation to the correct aminoacids that demonstrate strong internal binding and finally form rigid and functional polypeptide chains (proteins). Transcriptome to proteome transformation requires the m RNA binding by 40S subunit that provides also the eligible decoding region, whereas 60S subunit creates the substrate for peptide chain synthesis, development and progression by using the main ribosomal catalytic site (Sloan et al., 2017; Gościńska and Topf, 2020). At the end of these reactions, naive proteins are exported to the cytoplasm. Besides the appropriate regulation of HR over activation, the reversal process acting as a translation—suppressing mechanism is also of great importance. HRs disassembly that leads to protein synthesis suppression is mediated by their dimerization into hibernation complexes (Matzov et al., 2019; Slimane et al., 2020).

## Ribosome Functional Specialization-Dysfunction

Based on extensive molecular/structural analyses of the cellular micro-environment, there is strong evidence that ribosome heterogeneity exits and affects crucially the post-transcriptional and translational procedures (Shi and Barna, 2015; Parks et al., 2018; Ferretti and Karbstein, 2019). Continuous regulation of gene expression is a critical process mediated by a variety of inter- and intracellular signaling transduction. rRNA and mRNA modifications (splicing) lead to specific protein construction securing its normal functionality. Cell differentiation is characterized by increased numbers of newborn ribosomes with specific structural and functional features (Jacobs et al., 1985; Shi et al., 2017). The idea of intracellular ribosome diversity is proposed by study groups that reported ribosome sub-populations with specific characteristics. Ribosome specialization modifies rRNA post-transcriptional protein production and diversity (Krogh et al., 2016; Simms et al., 2017).

Besides ribosome specialization, ribosome dysfunction -caused by genetic alterations—negatively affects normal cell translational machinery. Abnormal ribosome biogenesis mediated by specific point mutations is implicated in the onset of congenital human disorders (syndromes) including X-linked dyskeratosis congenita, Treater Collins syndrome, Diamond—Blackfan anemia, 5q-syndrome, Schwachman—Diamond syndrome, and also Cartilage hair hypoplasia (Narla and Ebert, 2010). According to specific molecular studies focused on the potential genetic mechanisms involved in these ribosomopathies, ribosomal haploinsufficiency leading to increased oxidative stress, hemolysis, aberrant translation, insufficient, altered maturation and final synthesis of ribosomal subunits are main causes of the corresponding abnormal phenotypes (Joslin et al., 2007; Pellagatti et al., 2008). Multi-mutational analyses have shown a broad landscape of alterations in genes that encode for ribosomal proteins including mainly the DKC1, TCOF1, SBDS, RPS14, RPS19, and RPL5 (Liu and Ellis, 2006). Interestingly, a main mechanism implicated in abnormal ribosome biogenesis and dysfunction refers to murine double minute 2 protein (MDM2)-p53 pathway (Fang et al., 2000; Dai et al., 2004). Because MDM2 is a negative regulator of p53 protein expression, aberrant ribosomal production and activity strongly binds and deactivates MDM2 leading to a p53 over-activation (Fumagalli et al., 2009). The role of abnormal ribosomal biogenesis and dysfunction in cancer development and progression is under investigation. Similarly, the role of ribosome alterations in the exogenous RNA signals, such as virus RNAs regarding their distinct mRNAs translational process remains under investigation (Nahand et al., 2020).

## SARS-CoV-2 Genomic- Protein Structure

SARS-Cov-2 virus belongs to lineage b of beta-CoVs demonstrating a strong phylogenetic similarity with BatCoVRaTG13 type (Li et al., 2005; Wu et al., 2020). The viral genome consists of a large non-segmented, positive-sense RNA molecule of approximately 30 kb. The corresponding RNA-dependent RNA-polymerase (Rd-Rp) is essential for replicating the virus in the cytoplasm of the target epithelial cells. Analyzing SARS-CoV-2 spherical virion’s structure (diam ∼100 nm), research groups have confirmed that there are four main proteins including the spike surface glycoprotein (S), the main or matrix protein (M), the envelope protein (E), and finally the nucleocapsid protein (NC), whereas a variety of non-structural proteins have been also identified. In fact, 16 non-structural proteins (NSP1–NSP16) that encode for the RNA-directed RNA polymerase, helicase, and other components required for virus replication and translation in target cell ribosome machinery have been reported, whereas the functional role of other seven accessory proteins (ORF3a–ORF8) remains under investigation (Chen and Zhong, 2020; da Silva et al., 2020; Finkel et al., 2021). S glycoprotein projections -consisting of two subunits S1/S2- provide a unique crown-like formation (corona) on virion’s surface. Concerning their functional role, S1 represents the main receptor-binding domain (RBD), whereas S2 is involved in the virus-cell membrane fusion mechanism interacting with proteases, such as furin, thrypsin, cathepsin, or serino-protease TMPRSS2 (Coutard et al., 2020; Lukassen et al., 2020; Walls et al., 2020). Novel molecular and structural/crystallographic analyses have focused on a specific cell membrane receptor—the human angiotensin-converting enzyme 2 (hACE2)—which is the main target-functional receptor for SARS-CoV-2 cell attack, attachment, and entry that leads to S1 and S2 subunits activation (Ge et al., 2013). Interestingly, h ACE2 mediated SARS-CoV-2 cell entry seem to trigger a variety of intracellular signaling pathways, including hypoxia regulatory molecules (Tsiambas et al., 2020b). COVID-19 demonstrates aggressive clinic-pathological profiles in significant subsets of the infected patients—especially in males—and for this reason the role of chromosome X hosting the hACE2 gene (band Xp22.2) seems to be critical (Tsiambas et al., 2020a).

## LNP-Based v-mRNA Cell Entry

A variety of anti-COVID-19 vaccine types are under development in different phases, evaluation or even some of them have already completed these stages, validated and approved by FDA, EMA and other national health authorities for *in vivo* use in general population, accelerating their production and distribution process (Lee et al., 2020). Conventional vaccine platforms are based on the use of weakened or inactivated pathogens including whole virus or protein/peptide subunits, and replicating or non-replicating viral vectors. Besides them, DNA plasmid and innovative nucleoside-modified v-mRNA—based vaccines encapsulated within nanoparticles -specifically lipid ones (LNPs) by implementing electrostatic interactions—are now authorized for massive vaccination programs (Chung et al., 2020). A study observed that intra-dermal/muscular injection of these vaccines demonstrates extended duration of the mRNA expression compared to intravenous/subcutaneous injection (Pardi et al., 2015). The second step in providing targeted nucleic vaccines should be a long-term monitoring of the vaccinated populations in order to improve safety, efficacy, and immune response.

Despite the rapid design, development, validation initial approval as conditional marketing authorisation for massive production and vaccination of general population worldwide, there are some scientifically crucial parameters recognized at the cell and molecular level that should be clarified. According to BioNTech/Pfizer/Moderna Pharmaceutical Corporations, mRNA based vaccines import an encapsulated LNPs SARS-CoV-2 genomic sequence fragment inside the cells in order to produce a mimicked viral S protein. In fact, this novel vaccine platform includes the specific mRNA information for encoding the whole trimeric form of S protein and also the corresponding RBD region (mRNA- BNT162b2) or stabilized S protein (m RNA-1273) (Jackson et al., 2020). Concerning the mechanism of intracellular action, the v mRNA platform does not interact with or modify the host cell DNA because typically there is no integration into it. Cell entry of encapsulated v-mRNA in LNPs by penetrating the membrane is followed by genomic sequences release into cytoplasm targeting the ribosomal machinery and continuously the rough endoplasmic reticulum. Alternative mRNA vaccine technology strategies provide a self-amplification mechanism of the corresponding strands for producing and exporting a significantly larger number of nAbs (Pardi et al., 2018; Fuller and Berglund, 2020). Complete SARS-CoV-2 mRNA strand fragment translation leads to S protein neutralizing antibody (nAb) multiple copies production, motivating human immune system to respond protectively against the SARS-CoV-2 infection by sensitizing specific CD4 and CD8 T cells for exposing a high immunogenic activity (Wang et al., 2020). It is also important to be mentioned that host cell-mediated post-translational modifications (PTMs)—including predominantly glycosylation and phosporylation—are crucial for the final proteins’ functionality in viral infections (Kumar et al., 2020a). Especially, in SARS-CoV-2 RNA cell entry, differentially expressed human glycogens have been recently detected (Oommen et al., 2021). In rare cases, potentially altered, defective S protein nAbs’ production—as a result of the previous referred modifications—could not be excluded.

## mRNA COVID-19 Vaccines Potential Intracellular Interactions

Besides HR-mediated post-transcriptional, translational mechanism, there are some points of view with potentially high molecular importance. Naked mRNA strands are fragile, unstable *in vivo* and broken down in the intracellular microenvironment. In fact, cells have developed specific RNA- mRNA energy depended mechanisms for mediating mRNA transport from the transcription to translation sites or in the case of extracellular ones to the ribosome machinery only (Kiebler and Bassell, 2006). There is a variety of cytoplasmic half-life duration regarding transcripts of different origin. Degradation of these mRNAs ranges between a few minutes to some days (Yang et al., 2003; Ben-Ari et al., 2010). LNP delivery formulation prevents a potential nuclease degradation of the corresponding genomic strands. Automated, unpredictable LNPs rupture could release some strands inside the cytoplasm as naked viral mRNA sequences. These free mRNA “remnants” should be targets for human secretory ribonucleases (hRNases) –especially hRNase5 (angiogenin)—involved in the metabolism of intracellular RNAs (Lee et al., 2019). HRNase 5—a member of the hRNaseA superfamily—demonstrates the ability to be internalized into the cells regulating RNA species of different origin (extracellular viral RNAs). Furthermore, the molecule is involved in angiogenesis and neovascularuzation under the influence of Plexin-B2 (Yu et al., 2017). HRNase5 presents a high concentration level in the nucleoli after its translocation into the nucleus involved also in the ribosome assembly. It is also implicated in rRNA and mRNA transcription. Concerning viral infections (i.e., syncytial virus in respiratory tract) it provides tRNA cleavage increasing small tiRNA fragments that are essential for viral replication (Wang et al., 2013). Although LNPs—based mRNA delivery system seems to be more sophisticated and effective—compared to polymers or oil-in-water emulsions—are depended on temperature limitations (Blakney et al., 2020). This is an important and challenging parameter. Increased temperature probably disorganizes the LNP-mRNA complex affecting its proper entry to cytoplasm. Furthermore, LNPs size and early escape from endosomal activity are critical factors for a high level protein synthesis (Yanez Arteta et al., 2018; Sayers et al., 2019). So, naked SARS-CoV-2 mRNA strands -as a result of a potentially defective LNP inside the cytoplasm even for a very short period of time -could potentially be a target for RNases driving the cell to promote viral post-transcriptional amplification of this specific v-mRNA fragment or destroy it. This mechanism has been detected and analyzed for Influenza A RNA-viruses mediated seasonal respiratory infections (epidemics). A study group reported that ERI1 exonuclease binding to histone mRNA provided Influenza A viral transcription (Declercq et al., 2020).

Concerning v-mRNA influence in host cell genomic mechanism, some recently published studies showed severe alterations affecting normal ribosome functional activity. SARS-CoV-2 antagonizes crucial intra-cellular procedures by disrupting splicing, translation mechanisms and also protein recruitment and motivation, cytokine-interferon signaling, and ubiquitin mediated proteolysis in order to suppress host cell defenses (Banerjee et al., 2020). Precursor mRNA splicing mediated by spliceosome—a vast RNA/protein complex- is a crucial mechanism for regulating gene expression. Removal of introns (non-coding loci) combined with exons splicing (matching) leads to mRNA maturation (Bessa et al., 2020). Alternative splicing mechanism provides a transcript diversity that leads to alternative protein patterns (isoforms) (Wang et al., 2008; Blencowe, 2017; Kim et al., 2018). These important procedures seem to be affected by SARS-CoV-2 cell penetration. Splicing mechanism regarding the v-mRNA is an unexplored target for investigation. Studies analyzing human immunodeficiency virus type 1 (HIV-1) RNA transcripts and the corresponding protein products have revealed spliced mRNA forms (Truman et al., 2020). Potential spliced mRNA variations combined with mutations could negatively modify the efficacy of the current commercially available mRNA based vaccines. Multiple novel virus variant strains have detected harboring new mutations increasing infectivity/transmission rates (N501Y, HV69-70del, D614G, Y144del, and T716l) (Gu et al., 2020). N501Y mutation (A23063T substitution) affects the RBD region, enhancing the SARS-CoV-2 binding affinity to the selective receptor hACE2. Concerning the D614G mutation, there are controversial data regarding its role in v-mRNA mediated immunogenic response rates. A sturdy group analyzing D614G on pseudovirus substrate showed no evidence of potential neutralization escape regarding the current vaccines (Weissman et al., 2021). Similarly, another recently published paper explored the role of the British variant, which although previously infrequent D614G mutation is now globally dominant (Conti et al., 2021). They also suggested that maybe this mutation does not create a serious vaccine problem. In contrast to British variant, other recently isolated spike protein mutant patterns (South African and Brazilian) seem to influence partially the effectiveness of the first generation v-mRNA vaccines in sub-groups of individuals that demonstrate a level or resistance (Tada et al., 2021). Additionally, recent detection of recurrent spot deletions in the S- glycoprotein is a very important molecular issue implicating in neutralizing antibodies escape and potentially reduce the efficacy of the vaccines and anti-SARS-CoV-2 monoclonal antibodies (McCarthy et al., 2021). Another study group reported diminished neutralization potency *in vitro* against some emerging variants (Chen et al., 2021). So, there is skepticism regarding the level of their effectiveness and the need for enriching them with new mutational panel for a successive intracellular production of the modified nAbs (Moore and Offit, 2021; Noor, 2021).

## Conclusion and Perspectives

Anti- SARS-CoV-2 strategies are based on a variety of vaccine platforms. Novel and promising v-mRNA-based vaccines is a modern approach for stimulating an enhanced and effective immunogenic response. They are characterized by rapid development and low-cost massive production procedures, free of viral pathogens, whereas very low temperatures (freezing status) conditions are obligatory by now in order LNP-mRNA complex to be secured and drastically effective inside the intracellular environment. Concerning the risk in interacting with the nuclear microenvironment (host DNA), it seems to be very low. In contrast, a recently published study reported the possibility for RNA viruses—including SARS-CoV-2—to interfere with the crucial p53 “guardian of the genome” nuclear phospoprotein function (Alzhanova et al., 2021). The precise period of time after vaccination characterized by high immunogenicity and the probability of vaccinated people to be able as carriers to spread the virus still remain unknown. Ribosome machinery is the crucial organelle-target for SARS-CoV-2 genome translational procedures essential for the proper S protein nAbs production and cellular export. Ribosome dysfunction, altered splicing mechanisms, critical mutations in a rapidly transformed phylogeographical mosaic pattern (Kumar et al., 2020b), ribonuclease activity and LNP fragility should be considered important parameters that potentially modify efficacy-safety of mRNA vaccines. New molecular hypotheses regarding the influence of SARS-CoV-2 RNA transcripts in ribosome machinery, but also in the mitochondria and their host ribosomes open horizons in understanding unexplored intracellular reactions (Singh et al., 2020). SARS-CoV-2 RNA/mRNA kinetics inside the cell microenvironment is a target for further research. Systematic monitoring after vaccination in a significant period of time is critical for detecting unexplored molecular and clinical features potentially triggered by altered intracellular mechanisms. New molecular knowledge provides an improved substrate for the next v-mRNA vaccine generation.

## Author Contributions

ET, AC, VP, and EK wrote the manuscript. NM, VR, AB, DP, NK, and AL were academic advisors. ET produced the figure (scheme). All authors contributed to the article and approved the submitted version.

## Conflict of Interest

The authors declare that the research was conducted in the absence of any commercial or financial relationships that could be construed as a potential conflict of interest.
